# HLA class I and II genotype of the NCI-60 cell lines

**DOI:** 10.1186/1479-5876-3-11

**Published:** 2005-03-04

**Authors:** Sharon Adams, Fu-Meei Robbins, Deborah Chen, Devika Wagage, Susan L Holbeck, Herbert C Morse, David Stroncek, Francesco M Marincola

**Affiliations:** 1 Immunogenetics Section, Department of Transfusion Medicine, Clinical Center, National Institutes of Health, Bethesda, Maryland, 20892, USA; 2 Developmental Therapeutics Program, Information Technology Branch, National Cancer Institute, Bethesda, Maryland, 20892, USA; 3 Laboratory of Immunopathology, National Institute of Allergy and Infectious Diseases, Rockville, USA

## Abstract

Sixty cancer cell lines have been extensively characterized and used by the National Cancer Institute's Developmental Therapeutics Program (NCI-60) since the early 90's as screening tools for anti-cancer drug development. An extensive database has been accumulated that could be used to select individual cells lines for specific experimental designs based on their global genetic and biological profile. However, information on the human leukocyte antigen (HLA) genotype of these cell lines is scant and mostly antiquated since it was derived from serological typing. We, therefore, re-typed the NCI-60 panel of cell lines by high-resolution sequence-based typing. This information may be used to: 1) identify and verify the identity of the same cell lines at various institutions; 2) check for possible contaminant cell lines in culture; 3) adopt individual cell lines for experiments in which knowledge of HLA molecule expression is relevant. Since genome-based typing does not guarantee actual surface protein expression, further characterization of relevant cell lines should be entertained to verify surface expression in experiments requiring correct antigen presentation.

## Background

A panel of sixty cancer cell lines of diverse lineage (lung, renal, colorectal, ovarian, breast, prostate, central nervous system, melanoma and hematological malignancies) was developed, characterized and extensively used by the National Cancer Institute's Developmental Therapeutics Program (NCI-60) since the early 90's as a screening tool for anti-cancer drug development [1]. This strategy [2-9]. yielded data about drug-related cytotoxicity for about 100,000 compounds. In addition, extensive functional characterization of the NCI-60 response to diverse biological or chemical stimulation has been accumulated [10-15]. Although originally developed for chemo-sensitivity testing, with the development of high-throughput analyses the NCI-60 panel has been broadly characterized for other biological applications [16-25]. Thus, patterns incidentally identified provided platforms for further investigations of mechanisms of tumorigenesis and cancer progression [5,6,26-30]. More recently, genomic DNA [24] and proteomics analyses have further characterized the profile of these cell lines [31]. The combined database provides the most comprehensive phenotyping of commonly accessible cancer cell lines offering correlative information about genetic, transcriptional and post-translational qualities. With growing interest in the identification of novel tumor antigens recognized by T cells as targets for antigen-specific immunization ([32], the NCI-60 could become an ideal tool for *in silico *discovery [33] ([34] and for tumor cell-specific T-cell reactivity testing [35]. For this purpose, accurate information about the extended human leukocyte antigen (HLA) phenotype of each cell line is necessary for the definition and validation of specific HLA/epitope combinations. Although antiquated and partial information about the HLA phenotype of some of the NCI-60 cell lines is available through the American Type Culture Collection (ATCC), Rockville, MD, no high-resolution information obtained by definitive sequence-based typing (SBT) has ever been published. Since T cell recognition of HLA-epitope complexes is narrowly restricted to unique combinations [36], this information is critical to select reasonable candidates for antigen-discovery choosing cell lines bearing HLA phenotypes most relevant to the disease population studied [37]. Accurate information about the HLA genotype of each cell line may, in addition, help their identification, validation and qualification among different laboratories excluding possible errors related to switching of cell lines or culture contamination. Therefore, we provide high-resolution SBT of the complete NCI-60 panel obtained from their original source: the National Cancer Institute's Developmental Therapeutics Program.

## Results and Discussion

### Previous knowledge of the HLA phenotype of NCI-60 cell lines

We reviewed and collected available information about the HLA phenotype of the NCI-60 cell lines, performed according to serological testing before submission to the ATCC (Table 1). The information was collected through the ATCC website: . Most cell lines had not been previously typed; the large majority of the cell lines from which such information is available had been developed from Caucasian patients. HLA typing was reported according to the old serologic nomenclature at a very low level of resolution. In addition, several reported typings did not match the present typing as shown in Table 2 and 3. This was the case for the colon carcinoma cell line HT29 that maintained a correct haplotype (with the exclusion of the HLA-Cw locus) but had a completely different second haplotype. The melanoma cell line SK-MEL-5 had an almost identical haplotype with the exception of one HLA-B allele originally typed as Bw16 (inclusive of the molecularly-defined alleles: B*38 and B*39), while the present typing was HLA-B*07. Another melanoma cell line SK-MEL-28 maintained a haplotype similar to the previously reported HLA-A11, -B40 but appeared to have lost an HLA-A allele (HLA-A26) compared with the original ATCC description. Finally, the multiple myeloma cell line RPMI 8226 was matched at one haplotype (HLA-A19, -B15 and -Cw2) but was totally discrepant at the second haplotype (HLA-A*6802, -B*1510 and -Cw*0304). The HLA typing of the other two previously typed cell lines was confirmed in the present study. Overall, in spite of the discrepancies in HLA typing observed between the previous and the present analyses, a resemblance was noted in the cell line genotype suggesting that mis-typing related to the low accuracy of serological methods might have been at the basis of the discrepancy rather than contamination or switching of the cell lines.

**Table 1:** Available information from the ATCC about the NCI-60 panel

Name	ATCC no.	Sex	Race	Tumor Type	ATCC HLA typing	Discrepant
BT-549	HTB-122	F	C	Breast CA		
HS 578T	HTB-126	F	C	Breast CA		
MCF7	HTB-22	F	C	Breast CA		
MDA-MB-231	HTB-26	F	C	Breast CA		
MDA-MB-435	HTB-129	F	C	Breast CA		
T-47D	HTB-133	F		Breast CA		
SF-268				CNS CA		
SF-295				CNS CA		
SF-539				CNS CA		
SNB-19				CNS CA		
SNB-75				CNS CA		
U251				CNS CA		
COLO 205	CCL-222	M	C	Colon CA		
HCC-2998				Colon CA		
HCT-116	CCL-247	M		Colon CA		
HCT-15	CCL-225	M		Colon CA		
HT29	HTB-38	F	C	Colon CA	**A1,3,B12,17 Cw5**	**Yes**
KM12				Colon CA		
SW-620	CCL-227	M		Colon CA		
MOLT-4	CRL-1582	M		Leukemia, ALL		
CCRF-CEM	CCL-119	F	C	Leukemia, ALL		
HL-60	CCL-240	F	C	Leukemia, APL		
K-562	CCL-243	F		Leukemia, CML		
SR	CRL-2262	M	C	Leukemia, LCIL		
LOX IMVI				Melanoma		
M 14				Melanoma		
SK-MEL-2	HTB-68	M	C	Melanoma		
SK-MEL-5	HTB-70	F	C	Melanoma	**A2,11, B40,Bw16**	**Yes**
SK-MEL-28	HTB-72	M		Melanoma	**A11,26, B40,DRw4**	**Yes**
UACC-62				Melanoma		
UACC-257				Melanoma		
RPMI 8226	CCL-155	M		MM	**Aw19, B15,37, Cw2**	**Yes**
A549/ATCC	CCL-185	M	C	NSCLC		
EKVX				NSCLC		
HOP-62				NSCLC		
HOP-92				NSCLC		
NCI-H23	CRL-5800	M	AA	NSCLC		
NCI-H226	CRL-5826	M		NSCLC		
NCI-H322M				NSCLC		
NCI-H460	HTB-177	M		NSCLC		
NCI-H522	CRL-5810	M	C	NSCLC		
IGROV1				Ovarian CA		
OVCAR-3	HTB-161			Ovarian CA		
OVCAR-4				Ovarian CA		
OVCAR-5				Ovarian CA		
OVCAR-8				Ovarian CA		
NCI/ADR-RES				Ovarian CA		
SK-OV-3	HTB-77	F	C	Ovarian CA		
DU-145	HTB-81	M	C	Prostate CA		
PC-3	CRL-1435	M	C	Prostate CA	**A1,9**	**No**
786-O				Renal CA		
A498	HTB-44	F		Renal CA		
ACHN	CRL-1611	M	C	Renal CA		
CAK1-1	HTB-46	M	C	Renal CA	**A9,B12,35**	**No**
SN12C				Renal CA		
TK-10				Renal CA		
UO-31				Renal CA		
RXF-393				Renal CA		

AA = African American; ALL = Acute Lymphoblastic Leukemia; APL = Acute promyelocytic leukemia; C = Caucasian; CA = Carcinoma; CML = Chronic Myelogenous Leukemia; CNS = Central Nervous System; F = Female; LCIL = Large Cell Immunoblastic Lymphoma; M = Male; MM = Multiple Myeloma; NA = Not Available; NSCLC = Non Small Cell Lung Cancer.

The information about the ATCC cell lines (Cell Lines with ATCC no.) was obtained accessing the following URL: . Additional information was obtained through the National Cancer Institute's Developmental Therapeutics Program URL: .

**Table 2:** Sequence-based typing of NCI-60 HLA class I Loci

Cell Line	ID	Tissue	A locus	B Locus	Cw Locus
BT-549	41292-D	Breast CA	N.R.	151701, 5501	030301, 07^a^
HS 578T	41293-D	Breast CA	03^a^, 24^a^	35^a^, 40^a^	030401, 04^a^
MCF7	41294-D	Breast CA	020101	18^a^, 44^a^	05^a^
MDA-MB-231	41296-D	Breast CA	0201, 0217	4002, 4101	020202, 17^a^
MDA-MB435	41297-D	Breast CA	110101, 240201	15^a^, 35^a^	030301, 04^a^
T47D	41298-D	Breast CA	3301	1402	0802
SF-268	41286-D	CNS CA	010101, 3201	0801, 4002	020202, 07^a^
SF-295	41287-D	CNS CA	010101, 2601	070201, 5501	03^a^, 07^a^
SF-539	41288-D	CNS CA	020101	08^a^, 35^a^	04^a^, 07^a^
SNB-19	41289-D	CNS CA	020101	18^a^	05^a^
SNB-75	41290-D	CNS CA	020101, 110101	35^a^, 39^a^	04^a^, 120301
U251	41291-D	CNS CA	020101	18^a^	05^a^
COLO 205	41299-D	Colon CA	01^a^, 02^a^	07^a^, 08^a^	070201, 07^a^
HCC-2998	41300-D	Colon CA	02^a^, 24^a^	3701, 400601	04^a^, 0602
HCT-116	41301-D	Colon CA	01^a^, 02^a^	18^a^, 4501	05^a^, 07^a^
HCT-15	41302-D	Colon CA	02^a^, 24^a^	08new, 350101	04^a^, 07^a^
HT29	41303-D	Colon CA	01^a^, 24^a^	35^a^, 440301	04^a^
KM12	41304-D	Colon CA	02new	70201	70201
SW-620	41305-D	Colon CA	02^a^, 24^a^	07^a^, 15^a^	070201, 07^a^
MOLT 4	41281-D	Leukemia, ALL	010101, 2501	18^a^, 570101	0602, 120301
CCRF-CEM	41282-D	Leukemia, ALL	N.R.	08^a^, 40^a^	030401, 07^a^
HL-60	41284-D	Leukemia, APL	10101	570101	0602
K-562	41280-D	Leukemia, CML	110101, 310102	18^a^, 40^a^	03^a^, N.R.
SR	41285-D	Leukemia, LCIL	02^a^, 03^a^	3701, 3901	0602, 120301
LOX IMVI	41315-D	Melanoma	110101, 2902	070201, 440301	070201, 1601
M 14	41316-D	Melanoma	110101, 240201	15^a^, 35^a^	030301, 04^a^
SK-MEL-2	41317-D	Melanoma	03^a^, 26^a^	35^a^, 38^a^	04^a^, 120301
SK-MEL-5	41319-D	Melanoma	020101, 110101	07^a^, 40^a^	030401, 070201
SK-MEL-28	41318-D	Melanoma	110101	4001	030401
UACC-62	41321-D	Melanoma	02^a^, 32^a^	39^a^, 44^a^	05^a^, 12^a^
UACC-257	41320-D	Melanoma	020101	18^a^, 44^a^	05^a^, 07^a^
RPMI-8226	41283-D	MM	3001, 6802	1503, 1510	020204, 030402
A549/ATCC	41306-D	NSCLC	2501, 3001	18^a^, 440301	120301, 1601
EKVX	41307-D	NSCLC	010101	3701	0602
HOP-62	41308-D	NSCLC	030101	07^a^, 44^a^	05^a^, 070201
HOP-92	41309-D	NSCLC	03^a^, 24^a^	27^a^, 470101	01^a^, 06^a^
NCI-H23	41312-D	NSCLC	8001	5001	0602
NCI-H226	41311-D	NSCLC	010101, 240201	07^a^, 39^a^	070201, 120301
NCI-H322M	41310-D	NSCLC	2902	440301	1601
NCI-H460	41313-D	NSCLC	24^a^, 68^a^	35^a^, 51^a^	03^a^, 15^a^
NCI-H522	41314-D	NSCLC	020101	44^a^, 5501	030301, 05^a^
IGROV1	41322-D	Ovarian CA	240201, 3301	4901	07^a^
OVCAR-3	41323-D	Ovarian CA	020101, 2902	070201, 5801	070201, 07^a^
OVCAR-4	41324-D	Ovarian CA	010101, 3201	0801, 4002	07^a^, 15^a^
OVCAR-5	41325-D	Ovarian CA	01^a^, 02^a^	08^a^, 44^a^	05^a^, 07^a^
OVCAR-8	41326-D	Ovarian CA	010101, 2501	570101	0602
NCI/ADR-RES	41295-D	Ovarian CA	010101, 2501	570101	0602
SK-OV-3	41327-D	Ovarian CA	03^a^, 68^a^	18^a^, 35^a^	04^a^, 05^a^
DU-145	41328-D	Prostate CA	030101, 3303	5001, 570101	0602
PC-3	41329-D	Prostate CA	010101, 240201	1302, 5501	01^a^, 06^a^
786-O	41330-D	Renal CA	030101	07^a^, 44^a^	05^a^, 070201
A498	41331-D	Renal CA	020101	0801	07^a^
ACHN	41332-D	Renal CA	2601	4901	07^a^
CAKI-1	41333-D	Renal CA	2301, 240201	3502, 440301	04^a^, 04new
SN12C	41334-D	Renal CA	03, 24new	07^a^, 44^a^	05^a^, 070201
TK-10	41335-D	Renal CA	3301	1402	0802
UO-31	41336-D	Renal CA	010101, 030101	07^a^, 14^a^	07^a^, 08^a^
RXF-393	41337-D	Renal CA	02^a^, 24^a^	1401, 44^a^	05^a^, 0802

Sequence-based typing for the HLA class I loci are reported with the highest degree of resolution. Non-resolved ambiguities are reported as two digit denominations with a superscript ^a ^as previously described 43. HLA typings divergent from those originally described in the ATCC database are reported in red. ID# refers to the HLA laboratory reference number. New alleles are indicated by the suffix new following the allele. N.R. – Ambiguity not resolved at the lower level of resolution.

**Table 3:** Sequence-based typing of NCI-60 HLA class II Loci

Cell Line	ID	Tissue	DRβ1 Locus	DQB1 Locus	DPB1 Locus
BT-549	41292-D	Breast CA	11^a^, 13^a^	030101, 060401	020102, 0401
HS 578T	41293-D	Breast CA	01^a^, 150101	050101, 0602	0401, 7801
MCF7	41294-D	Breast CA	03^a^, 15^a^	0201, 0602	020102, 0401
MDA-MB-231	41296-D	Breast CA	0701, 1305	0202, 030101	020102, 1701
MDA-MB435	41297-D	Breast CA	040501, 130101	0302, 0603	1301, 1901
T47D	41298-D	Breast CA	010201	050101	020102, 0401
SF-268	41286-D	CNS CA	03^a^, 04^a^	0201, 0302	0401, 0601
SF-295	41287-D	CNS CA	14^a^, 15^a^	050301, 0602	0401
SF-539	41288-D	CNS CA	030101, 12^a^	0201, 030101	010101, 0401
SNB-19	41289-D	CNS CA	030101	0201	0402
SNB-75	41290-D	CNS CA	0103, 11^a^	03^a^, 050101	0401, 0402
U251	41291-D	CNS CA	030101	0201	0402
COLO 205	41299-D	Colon CA	040101, 130101	0603	0401
HCC-2998	41300-D	Colon CA	11^a^, 16^a^	030101, 050201	0401
HCT-116	41301-D	Colon CA	N.R.	02new, 03new	030101, 0402
HCT-15	41302-D	Colon CA	03^a^, 14^a^	02^a^, 050301	010101, 0401
HT29	41303-D	Colon CA	0402, 0701	02^a^, 0302	0401
KM12	41304-D	Colon CA	040101	0302	1301
SW-620	41305-D	Colon CA	0103, 130101	050101, 0603	010101, 0401
MOLT 4	41281-D	Leukemia, ALL	07new, 12new	0202, 030101	20102
CCRF-CEM	41282-D	Leukemia, ALL	030101, 0701	0201, 0202	0401, 1301
HL-60	41284-D	Leukemia, APL	N.R.	030302	0401, 1301
K-562	41280-D	Leukemia, CML	03^a^, 04^a^	0201, 0302	0401, 0402
SR	41285-D	Leukemia, LCIL	01^a^, 160101	050101, 050201	0401
LOX IMVI	41315-D	Melanoma	0701, 150101	0202, 0602	0401, 110101
M 14	41316-D	Melanoma	040501, 130101	0302, 0603	1301, 1901
SK-MEL-2	41317-D	Melanoma	0402, 130101	030101, 0603	020102, 0401
SK-MEL-5	41319-D	Melanoma	040101, 130101	0302, 0603	030101, 1601
SK-MEL-28	41318-D	Melanoma	0404	0302	030101
UACC-62	41321-D	Melanoma	12^a^, 130101	030101, 0603	0401, 1401
UACC-257	41320-D	Melanoma	040101	030101, 0302	0401
RPMI-8226	41283-D	MM	030101, 0701	0201, 0202	010102, 1301
A549/ATCC	41306-D	NSCLC	0701, 110401	0202, 030101	N.R.
EKVX	41307-D	NSCLC	150101	0602	0401
HOP-62	41308-D	NSCLC	13^a^, 15^a^	06^a^, 06^a^	0402
HOP-92	41309-D	NSCLC	01^a^, 150101	050101, 0602	0401, 0402
NCI-H23	41312-D	NSCLC	130101	0603	1901
NCI-H226	41311-D	NSCLC	150101, 160101	050201, 0602	020102, 0401
NCI-H322M	41310-D	NSCLC	0701	0202	0401
NCI-H460	41313-D	NSCLC	01^a^, 04^a^	030101, 050101	N.R.
NCI-H522	41314-D	NSCLC	040101, 150101	03^a^, 0602	0401
IGROV1	41322-D	Ovarian CA	11^a^, 11^a^	03new	new, 0501
OVCAR-3	41323-D	Ovarian CA	080101, 080401	0402	020102, 0401
OVCAR-4	41324-D	Ovarian CA	030101, 040101	0201, 030101	0401, 1301
OVCAR-5	41325-D	Ovarian CA	030101, 040101	0201, 030101	0401
OVCAR-8	41326-D	Ovarian CA	0701, 150101	030302, 0602	020102, 1301
NCI/ADR-RES	41295-D	Ovarian CA	0701, 150101	030302, 0602	020102, 1301
SK-OV-3	41327-D	Ovarian CA	01^a^, 030101	0201, 050101	020102, 0401
DU-145	41328-D	Prostate CA	N.R.	030302, 050101	0401
PC-3	41329-D	Prostate CA	0701, 130101	0202, 0603	0401
786-O	41330-D	Renal CA	13^a^, 15^a^	06^a^, 06^a^	0402
A498	41331-D	Renal CA	030101	0201	010101
ACHN	41332-D	Renal CA	160101	050201	020102
CAKI-1	41333-D	Renal CA	0701, 110401	0202, 03^a^	020102, 1001
SN12C	41334-D	Renal CA	040101, 150101	03^a^, 0602	N.R.
TK-10	41335-D	Renal CA	010201	050101	0402
UO-31	41336-D	Renal CA	130201, 150101	0602, 0609	0402, 0501
RXF-393	41337-D	Renal CA	110101, 150101	030101, 0602	010101, 0401

Sequence-based typing for the HLA class II loci are reported with the highest degree of resolution. Non-resolved ambiguities are reported as two digit denominations with a superscript ^a ^as previously described [43]. HLA typings divergent from those originally described in the ATCC database are reported in red. ID# refers to the HLA laboratory reference number. New alleles are indicated by the suffix new following the allele. N.R. = Ambiguity not resolved at the lower level of resolution.

Overall, there was no evidence of contamination among the cell lines tested with clean homozygous or heterozygous combinations observed in all loci analyzed. SBT of HLA class I and HLA class II loci are reported in Table 2 and 3 respectively. Information about the HLA typing of the cell lines is also available through the Molecular Targets URL: . Approximately 17% of the cell lines (10 out of 58 including: T47D, SNB-19, U251, KM12, RPMI-8226, EKVX, NCI-H23, NCI-H322M, A498, ACHN and TK-10) exhibited a pseudo-homozygous pattern suggestive of complete loss of heterozygosity encompassing the HLA class I and HLA class II regions. This frequency is close to the loss of haplotype that we originally described for melanoma cell lines generated at the National Cancer Institute (Bethesda, MD) [38,39] and subsequently observed in other cancers [40,41]. We conclude that this is an unlikely representative of patients' homozygosity because complete HLA class I and II homozygosity is exceedingly rare in the population at large. To corroborate this statement, we analyzed 554 genomic DNA specimens from normal donors recently typed with the same technology in our laboratory. Genomic DNA for the normal donors was obtained from whole blood samples. Only 5 individuals were found to be truly homozygous for all HLA class I and class II loci for a frequency of 0.9%.

Overall, discrepancies between ATCC typings and the present typing or the unbalanced frequency of homozygosity could be related to accumulated genetic alterations between the cell lines since the time of their original expansion from the patient and should not be surprising.

A particular case was represented by the NCI/ADR-RES cell line which was previously believed to be an adriamycin derivative of the breast cancer cell line MCF-7. Subsequently, it was discovered not to be related to MCF-7, but it's derivation was unclear [42]. Karyotyping analysis suggested it was related to the ovarian cell line OVCAR-8. Subsequent DNA fingerprinting confirmed that both cell lines were generated from the same individual. HLA genotyping confirms this since the cell lines are indeed identical.

To avoid possible misinterpretations, a large number of alleles are not presented here with their definitive nomenclature but rather at a two digits level of resolution because some of the ambiguities could not be completely resolved by SBT as previously described [43]. However, more detailed information about individual cell lines can be obtained by contacting Sharon Adams directly at the HLA laboratory, Department of Transfusion Medicine, Bethesda, MD. As previously described [43], it is possible to resolve most of these ambiguities using various methods including sequence-specific primer PCR or pyro-sequencing [44]. If necessary in the future, the NIH HLA laboratory may assist in further characterization of individual HLA alleles. Another caveat is that the identification of HLA alleles at the genomic level does not necessarily correspond to surface expression of their protein products since various abnormalities in transcription, translation and assembling could influence the surface expression of HLA molecules [39,45,46].

Finally, several new alleles were identified (referred to in the tables as new, for which a nomenclature is pending; in detail KM12 HLA-A*02new = Genebank Accession # AY918166; SN12C HLA-A*24new = # AY918167; CAKI-1 HLA-Cw04new = # AY918170). Information regarding the sequence of these alleles could be obtained by directly contacting the HLA laboratory, Department of Transfusion Medicine, Bethesda, MD.

## Materials and Methods

### Cell Lines

Genomic DNA from the NCI-60 cell line anticancer drug discovery panel was obtained from SH of the National Cancer Institute Developmental Therapeutics Program (Bethesda, MD). Cells were grown in RPMI 1640 supplemented with 10% fetal bovine serum and 5 mM L-glutamine.

### DNA Isolation

Genomic DNA was isolated from peripheral blood using the Gentra PUREGENE isolation kit (Gentra Systems, Minneapolis, MN, USA). The DNA was re-suspended in Tris HCl buffer (pH 8.5) and the concentration was measured using a Pharmacia Gene Quant II Spectrophotometer. The DNA was then stored at -70°C until testing.

### Sequence-Based Typing (SBT)

HLA class I loci sequence-based typing (SBT) was performed as previously described ([43]. The primary PCR amplification reaction produced a 1.5 kb amplicon encompassing exon 1 through intron 3 of the HLA class I locus. All reagents necessary for primary amplification and sequencing were included in the HLA-A, HLA-B and HLA-C alleleSEQR Sequenced Based Typing Kits (Atria Genetics, Hayward, CA, U.S.A.). The primary amplification PCR products were purified from excess primers, dNTPs and genomic DNA using ExoSAP-IT (American Life Science, Cleveland, OH, U.S.A.). Each template was sequenced in the forward and reverse sequence orientation for exon 2 and exon 3 according to protocols supplied with the SBT kits. Excess dye terminators were removed from the sequencing products utilizing an ethanol precipitation method with absolute ethanol. The reaction products were reconstituted with 15 μl of Hi-Di™ Formamide (PE Applied Biosystems / Perkin-Elmer, Foster City, CA, U.S.A.) and analyzed on the ABI Prism* 3700 DNA Analyzer with Dye Set file: Z and mobility file: DT3700POP6 [ET].
